# Supplementary material for: Species-specific metabolic reprogramming in human and mouse microglia during inflammatory pathway induction
Source: Nat Commun. 2023 Oct 13;14:6454. doi: 10.1038/s41467-023-42096-7 (PMC10575978; doi:10.1038/s41467-023-42096-7)
Supplement: Supplementary file 1 — Supplementary Information [file 41467_2023_42096_MOESM1_ESM.pdf]

SUPPLEMENTARY INFORMATION

Species-specific metabolic reprogramming in human and mouse microglia during inflammatory pathway induction

Authors

Angélica María Sabogal-Guáqueta[1,*], Alejandro Marmolejo-Garza[1,2*], Marina Trombetta Lima[1,2], Asmaa Oun[1], Jasmijn Hunneman[1], Tingting Chen[1], Jari Koistinaho[3,4] Sarka Lehtonen[3], Arjan Kortholt[5,6], Justina C. Wolters[7], Barbara M. Bakker[7], Bart J.L. Eggen[2], Erik Boddeke[2], Amalia Dolga[1]

[1.] Department of Molecular Pharmacology, Faculty of Science and Engineering, Groningen Research Institute of Pharmacy, Behavioral and Cognitive Neurosciences (BCN), University of Groningen, Groningen, the Netherlands

[2.] Department of Biomedical Sciences of Cells & Systems, section Molecular Neurobiology, Faculty of Medical Sciences, University of Groningen, University Medical Center Groningen, Groningen, the Netherlands

[3.] A.I. Virtanen Institute for Molecular Sciences, University of Eastern Finland, P.O. Box 1627, 70211 Kuopio, Finland.

[4.] Neuroscience Center, Helsinki Institute for Life Science, University of Helsinki, Haartmaninkatu 8, 00290 Helsinki, Finland

[5.] Department of Cell Biochemistry, University of Groningen, Groningen, the Netherlands

[6.] YETEM-Innovative Technologies Application and Research Centre Suleyman Demirel University, Isparta, Turkey.

[7.] Laboratory of Pediatrics, Section Systems Medicine of Metabolism and Signaling, Faculty of Medical Sciences, University of Groningen, University Medical Center Groningen, Groningen, the Netherlands

*These authors contributed equally

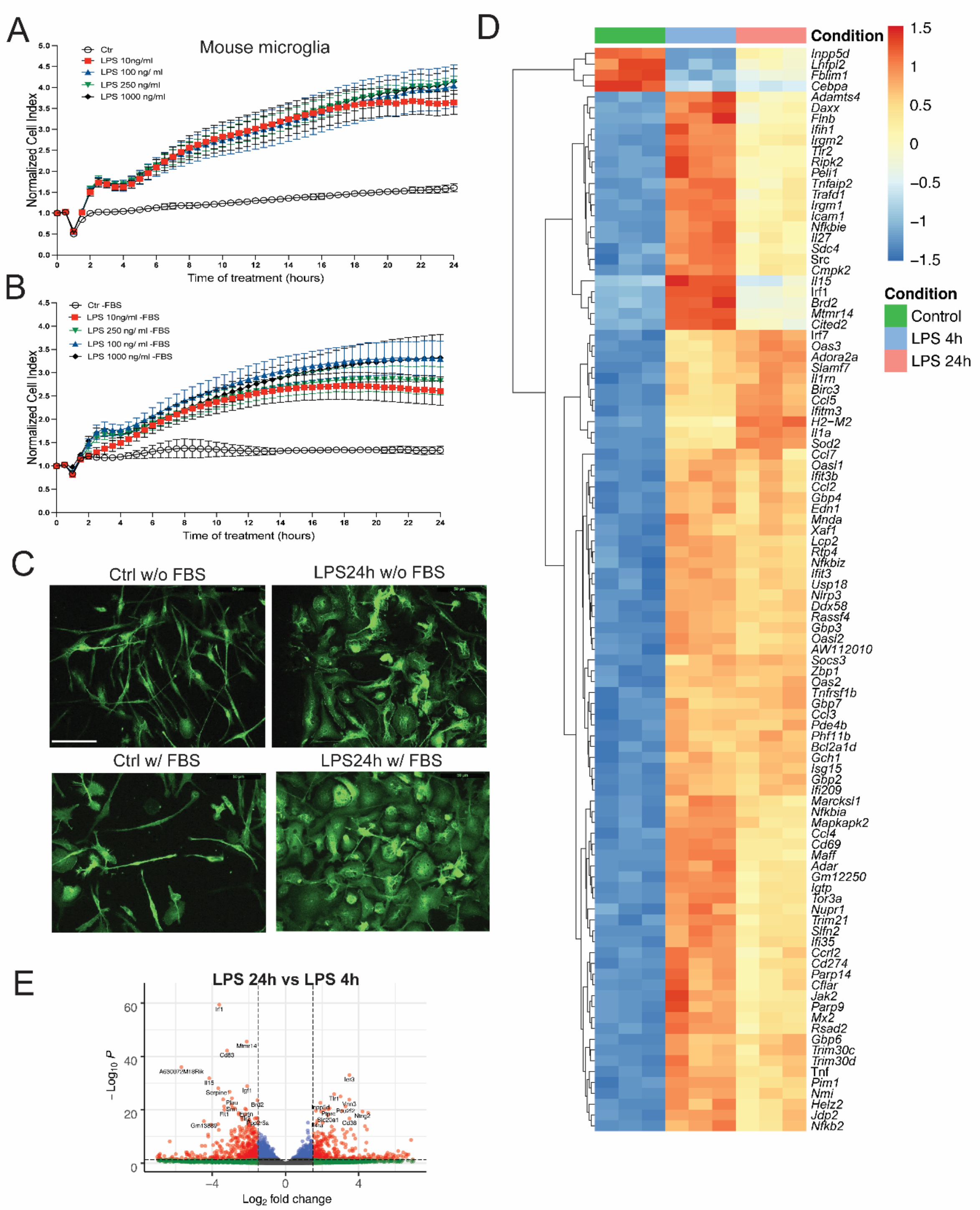

Supplementary Figure 1. LPS response to mouse microglia. A, B. Mouse cells were seeded in 96-well E-plates at a density of 15,000 cells/well and monitored by a real-time impedance-based xCELLigence system. Mouse microglia or iMGL cells were challenged with increasing concentrations of LPS (10-1000ng/ml) in the absence (A) or presence (B) of serum for 24hr. C. IBA1 immunostaining of control and LPS-treated mouse microglia with and without FBS. Magnification 20x. Scale bar: 50µm. D. Heatmap depicting the top 100 most significant differentially expressed genes of 4h LPS treatment compared with the control. Color key corresponds to row Z-score. E. Volcano plot depicting fold changes and $-Log_2$ of the adjusted *p* value per gene comparing responses against LPS 24h and 4h. In panel S1A, LPS 10ng/ml, 100ng/ml, and 1000ng/ml, N=6 technical replicates and LPS 250ng/ml, N=5 of a representative experiment which was repeated 3 times with similar results. In panel S1B, LPS 10ng/ml, 100ng/ml, and 250ng/ml, N=6 technical replicates and LPS 1000ng/ml, N=5 of a representative experiment which was repeated 3 times with similar results. For panel S1D, N=3 different animals per condition.

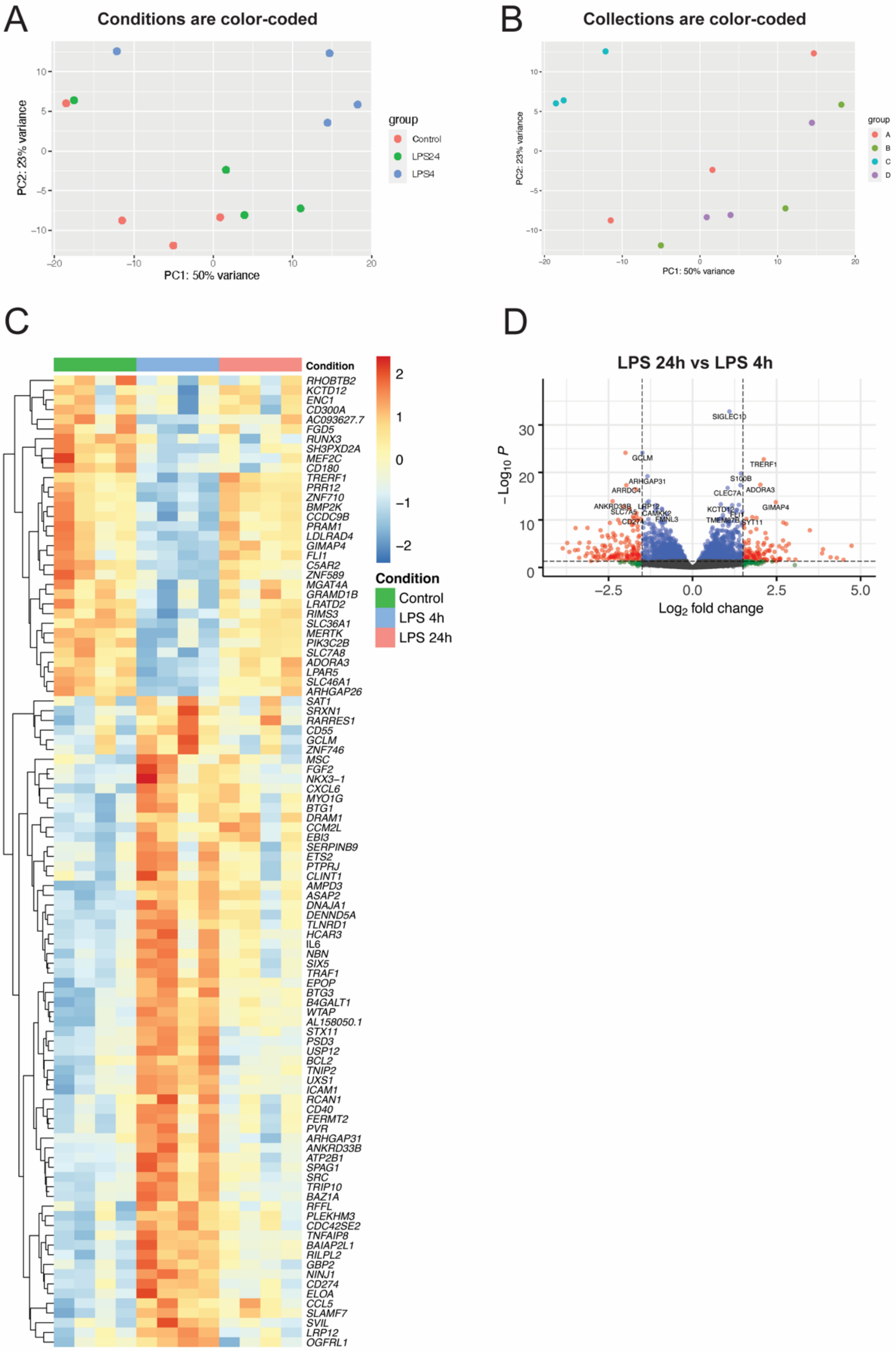

Supplementary Figure 2. Human iMGLs response to LPS

A, B. Principal Component Analysis (PCA) of the transcriptomes of untreated, and LPS-treated iMGLs for 4h and 24h. In (A), samples are colored by condition. In (B), samples are colored by collection/biological replicate. C. Heatmap depicting the top 100 most significant differentially expressed genes of 4h LPS treatment compared with the control. Color key corresponds to row Z-score. **D**. Volcano plot depicting fold changes and -Log_2 of the adjusted *p* value per gene comparing responses against LPS 24h and 4h. N=4 independent experiments per condition.

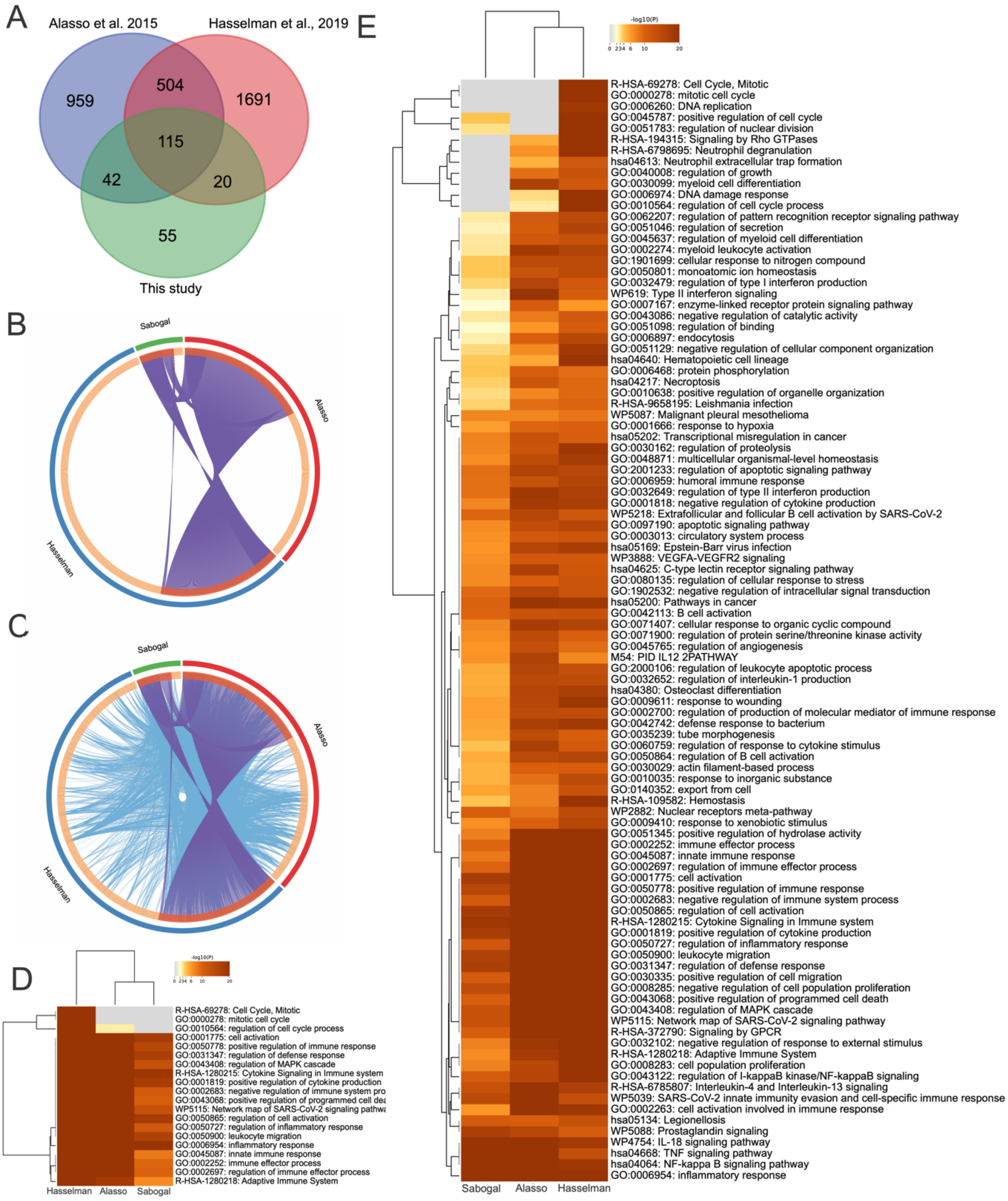

Supplementary Figure 3. Comparison of the transcriptomes of LPS-treated iPSC-derived human microglia-like cells from this study with other iPSC-derived microglia models.

A. Venn diagram with the overlap of DEGs of short-term LPS-treated iPSC-derived microglia-like cells from the study of Alasoo and colleagues, Hasselman and this study. B. Circos plot depicting the overlap in the lists of DEGs. On the outside, each arc represents the identity of each gene list, using the following color code: Blue, Hasselman; red, Alasso; green, this study. On the inside, each arc represents a gene list, where each gene member of that list is assigned a spot on the arc. Dark orange color represents the genes that are shared by multiple lists and light orange color represents genes that are unique to that gene list. Purple lines link the same gene that are shared by multiple gene lists. C. Circos plot built in the same way as (B). Blue lines link the genes that, although different, fall under the same ontology term (the term has to be statistically significantly enriched and with size no larger than 100). Blue lines indicate the amount of functional overlap among the input gene lists. D. Heatmap depicting the top 20 statistically enriched terms (GO/KEGG, canonical pathways, etc..) hierarchically clustered into a tree based on

Kappa-statistical similarities among their gene memberships. The term with the best p-value within each cluster is shown as its representative tem in the dendrogram. Heatmap cells are colored by their p-values, while cells indicate the lack of enrichment for that term in the corresponding gene list. E. Heatmap depicting the top100 statistically enriched terms in a similar fashion to (D).

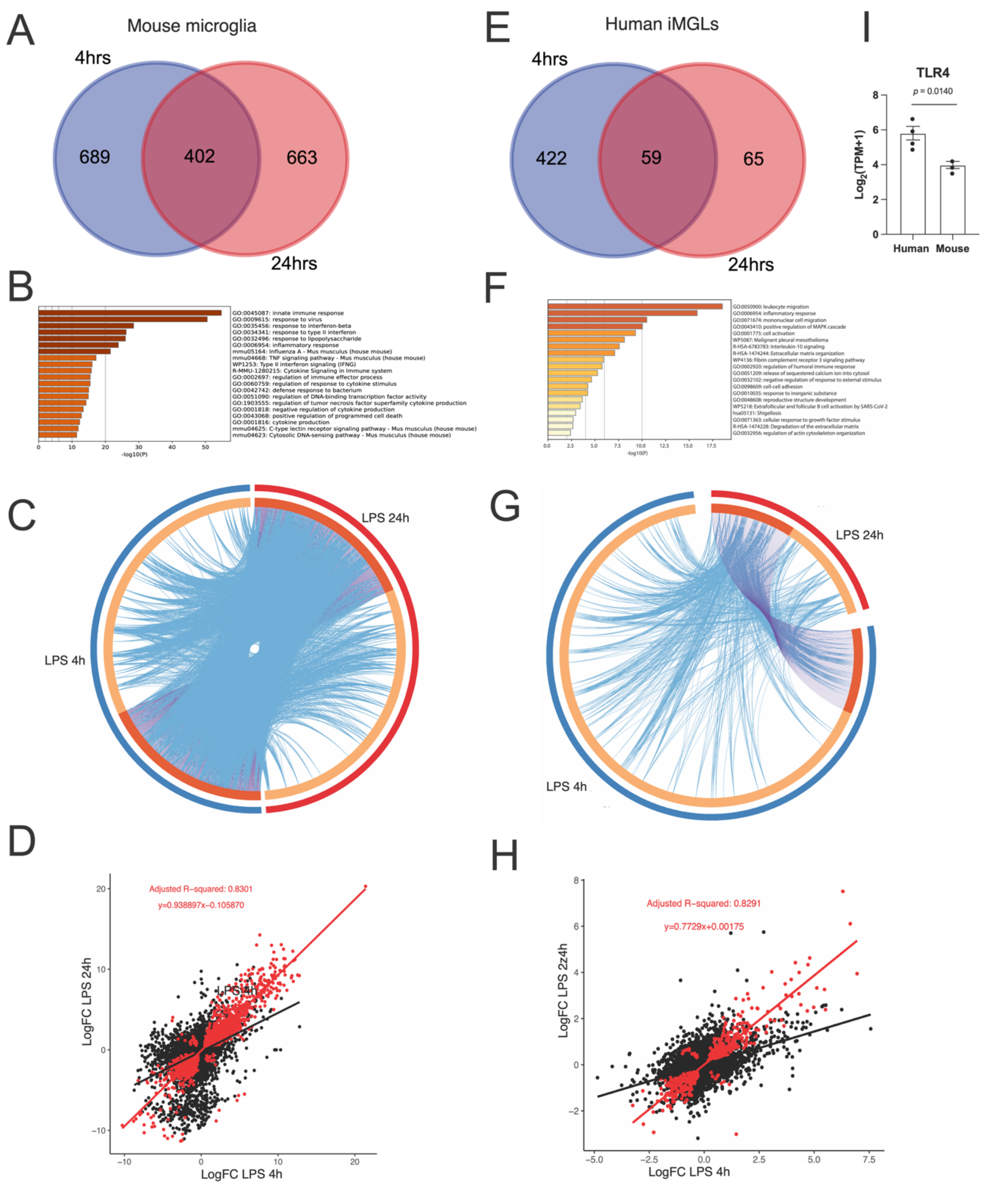

Supplementary Figure 4. Cross-species comparison of transcriptomic responses against LPS over time. A, E. Overlap of DEGs in mouse microglia (A) and human iMGLs (E) at 4 and 24 hours after LPS stimulation. B, F. Top enriched biological processes after performing GSEA with the metascape tool with the overlapped gene lists for mouse microglia (B) and human iMGLs (F). C, G. Circos plot depicting how genes from the lists of DEGs overlap for mouse microglia (C) and human iMGLs (G) for the two timepoints. On the outside, each arc represents the identity of each gene list. On the inside, each arch represents a gene list, where each gene member of that list is assigned a spot on the arc. Dark orange color represents the genes that are shared by multiple lists and light orange color represents genes that are unique to that gene list. Purple lines link the same gene that are shared by multiple gene lists (notice a gene that appears in two gene lists will be mapped once onto each gene list, therefore, the two positions are purple linked). Blue lines link the genes, although different, fall under the same ontology term (the term has to be statistically significantly enriched and with size no larger than 100). The greater the number of purple links and the longer the dark orange arcs imply greater overlap among the input gene lists. Blue links indicate the amount of functional overlap among the input gene lists. D, H. Scatterplots of the genes that were differentially

regulated by LPS 4h and 24h treatment (red dots) with an adjusted *p*<0.05 in mouse microglia (D) and human iMGLs (H). A linear model was fitted among the genes that were differentially regulated and the R squared, and the equation of the line are depicted within the scatterplot. I. Gene expression of the *Tlr4* and *TLR4* genes depicted by the normalized measure of TPMs per species. N= 4 human independent experiments and N=3 different animals. p=0.014 by unpaired two-tailed t-test.

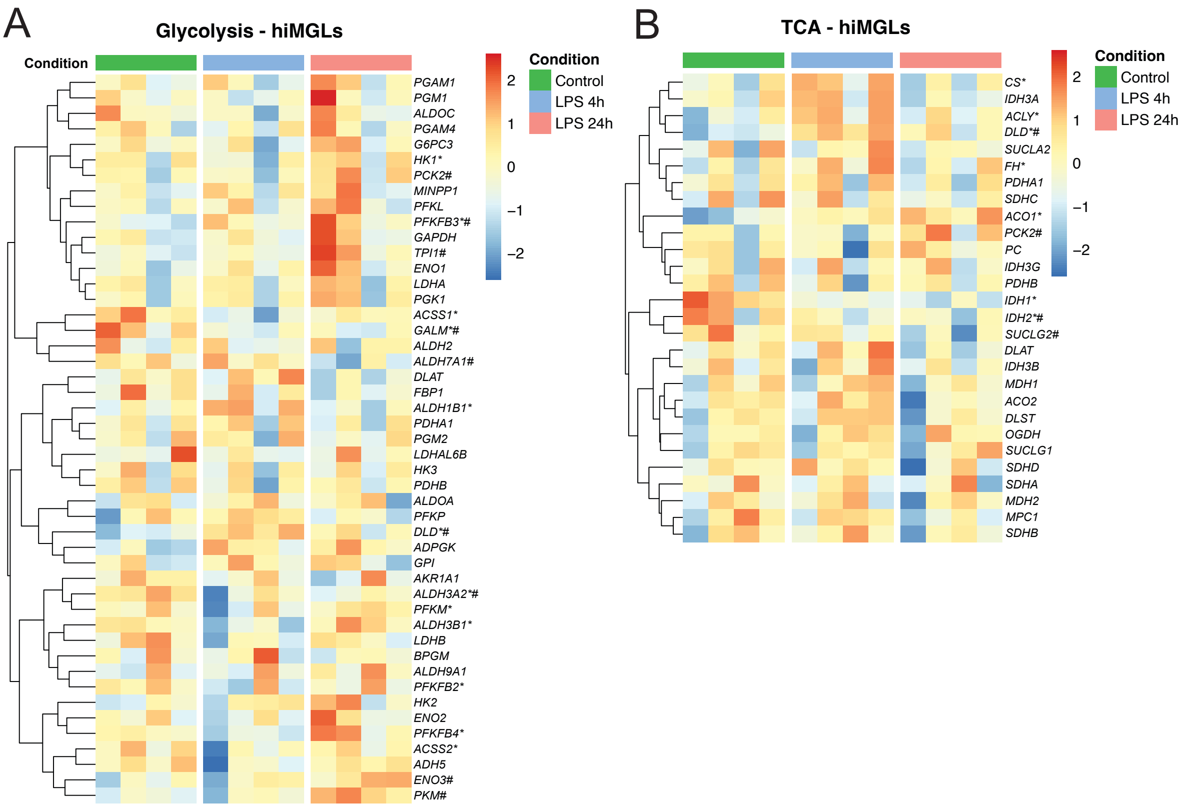

Supplementary Figure 5. Transcriptomic changes in the glycolytic and TCA pathways in LPS-treated human iMGLs. A, B. Hierarchically clustered heatmaps depicting gene expression changes in the glycolytic pathway (A) and in the TCA pathway (B). Color key corresponds to row Z-score. * denotes *padj* <0.05 LPS 4h versus Control, # denotes *padj* <0.05 LPS 24h versus Control and these values can be found in Supplementary Datasets. N=4 independent experiment per condition.

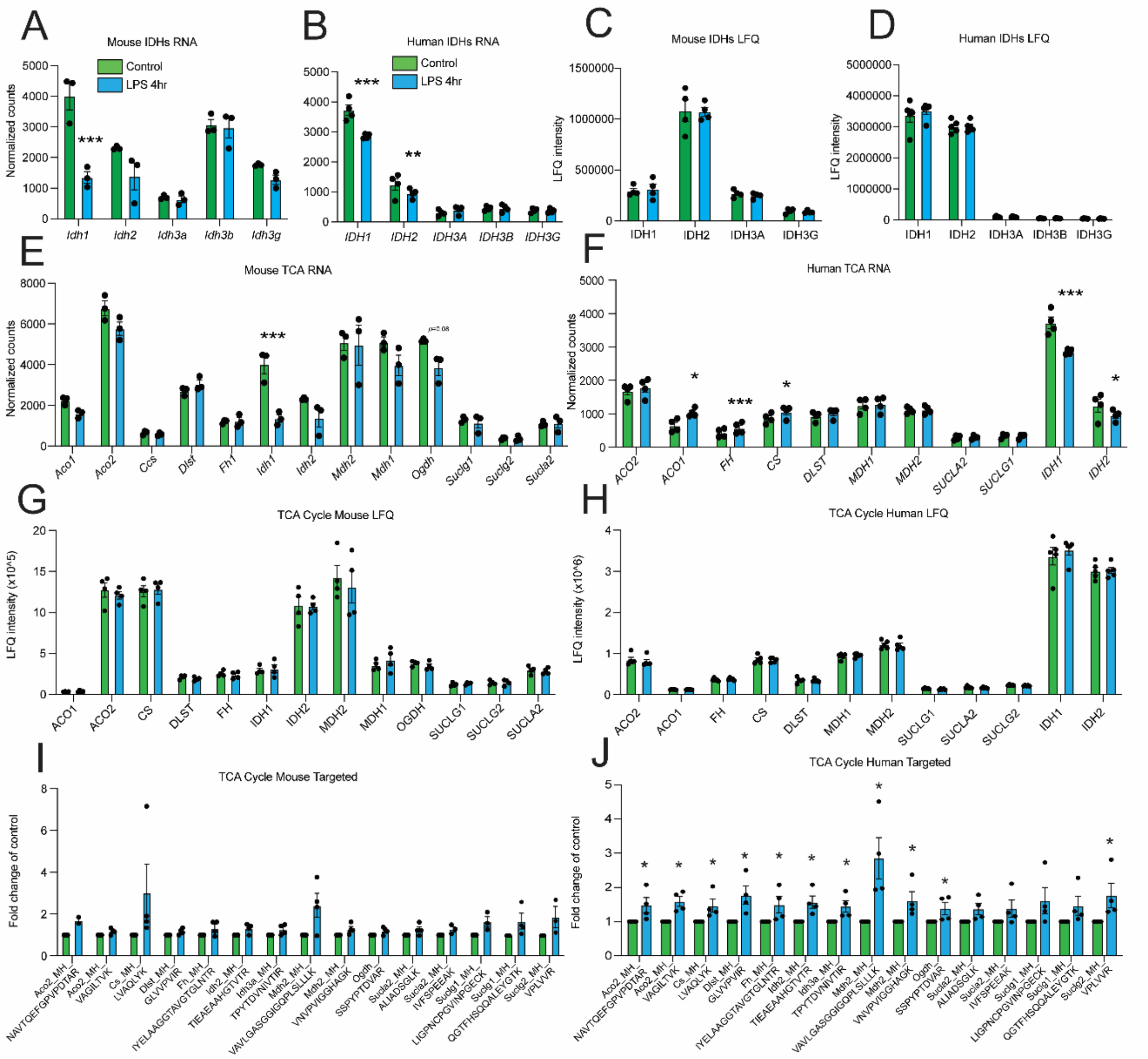

Supplementary Figure 6. Transcriptomic and proteomic characterization of TCA cycle enzymes in mouse microglia and human iMGLs. Mouse and human microglial cells were in vitro stimulated with LPS 250ng/mL and collected 4 hours after stimulation for proteomic analysis by LC-MS/MS. A,B. Normalized counts depicting transcript abundance of genes that code for Isocitrate dehydrogenase enzymes in mouse (A) (*Idh1*, *p*=1.39E^7) and human (B) cells. C,D. Label-free quantitation values from proteomic analysis depicting relative abundance of *IDH1* (*p*=0.00039), *IDH2* (*p*=0.025), and IDH3A and IDH3B in mouse (C) and human (D) cells. E,F. Normalized counts depicting transcript abundance of genes that code for TCA cycle enzymes in mouse (E) and human (F) cells (ACO1, *p*=0.00065; FH, *p*=0.00038, CS, *p*=0.047). G,H. Label-free quantitation values depicting relative abundance of TCA cycle enzymes in mouse (I) and human (J) cells. I,J. Proteomic abundances of TCA cycle enzymes measured by LC-MS/MS with internal standards. (I) Depicts plots of relative abundances on mouse microglia and (J) depicts human cells (ACO2, for first peptide *p*=0.03, second peptide *p*=0.014; CS, *p*=0.03; DLST, *p*=0.018; FH, *p*=0.04; IDH2, *p*=0.014; IDH3A, *p*=0.018; MDH2, first peptide *p*=0.022, second peptide *p*=0.03; SUCLA2, *p*=0.045; SUCLG2, *p*=0.031). For all panels, data are presented as mean ± S.E.M.. Every biological replicate is depicted as a dot. For mouse RNAseq N=3 different animals, for human RNAseq N=4 independent experiments, for mouse proteomics N=4 different animals and for human label free proteomics N=5 independent experiments, for human targeted proteomics N=4 independent experiments. For RNAseq data *p* values were determined by the Wald test and multi testing corrected in DESEQ2. For LFQ proteomic data, *p* values were determined by unpaired two-tailed t-test and for targeted proteomic data, p values were determine by paired one-tailed t-test. *p<0.05, **p<0.01, and ***p<0.001

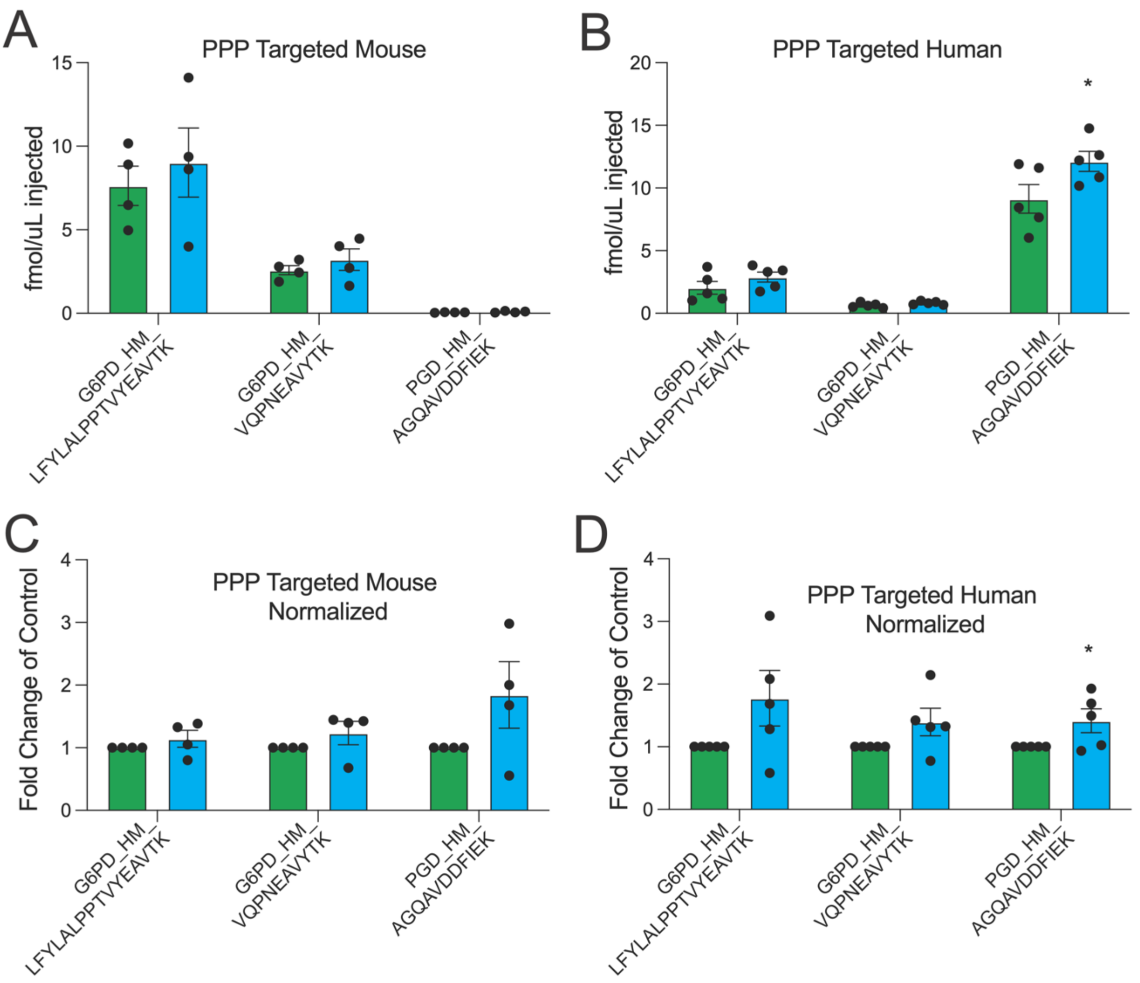

Supplementary Figure 7. Proteomic characterization of pentose-phosphate pathway enzymes in mouse microglia and human iMGLs. A, B. Absolute proteomic abundances of pentose phosphate pathway (PPP) enzymes measured by LC-MS/MS with internal standards. Mouse microglia are depicted in (A) and mouse iMGLs are depicted in (B) (PGD, *p*=0.032). C, D. Relative proteomic abundances of (A) and (B), respectively (PGD, *p*=0.031). For mouse targeted proteomics N=4 different animals and for human targeted proteomics N=5 independent experiments. All data are presented as mean ± S.E.M. *p* values were determined by paired two-tailed t-test. *p<0.05.

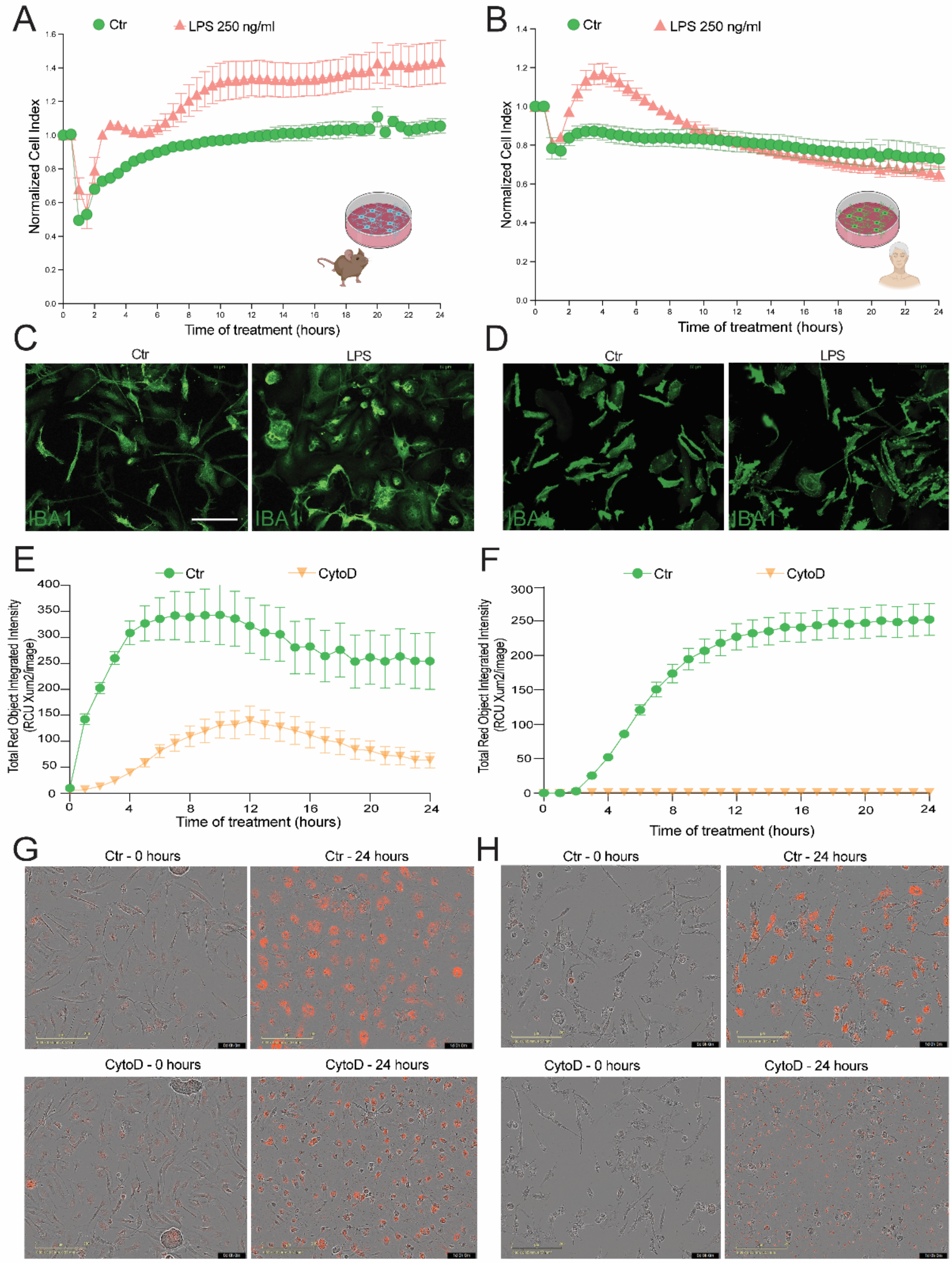

Supplementary Figure 8. Differential response to LPS in mouse and human microglia. A. Mouse and B. iMGL cells were seeded in 96-well E-plates at a density of 15,000 cells/well and monitored by a real-time impedance-based xCELLigence system. Mouse microglia or iMGL cells were challenged with LPS (250 ng/ml) for 24hr. Morphological alterations of microglia were visualized by immunostaining with Iba-1 antibody in mouse C and in D iMGL cells. Magnification 20x. Scale bar: 50µm. Phagocytic activity of mouse E and human F differentiated iPSC microglia in the Incucyte system displaying phagocytosis reagent pHrodo red *E. coli* BioParticles being engulfed by cells vs cells treated with the inhibitor Cytochalasin D (CytoD) with its corresponding time-lapse curves and representative pictures at time 0 and 24hr after the addition of the particles and phagocytic activity inhibitor. Magnification 20x. Scale bar: 200µm. For all panels, N=6 technical replicates of a representative experiment which was repeated 3 times with similar results. Figures S8A and S8B were made with Biorender.

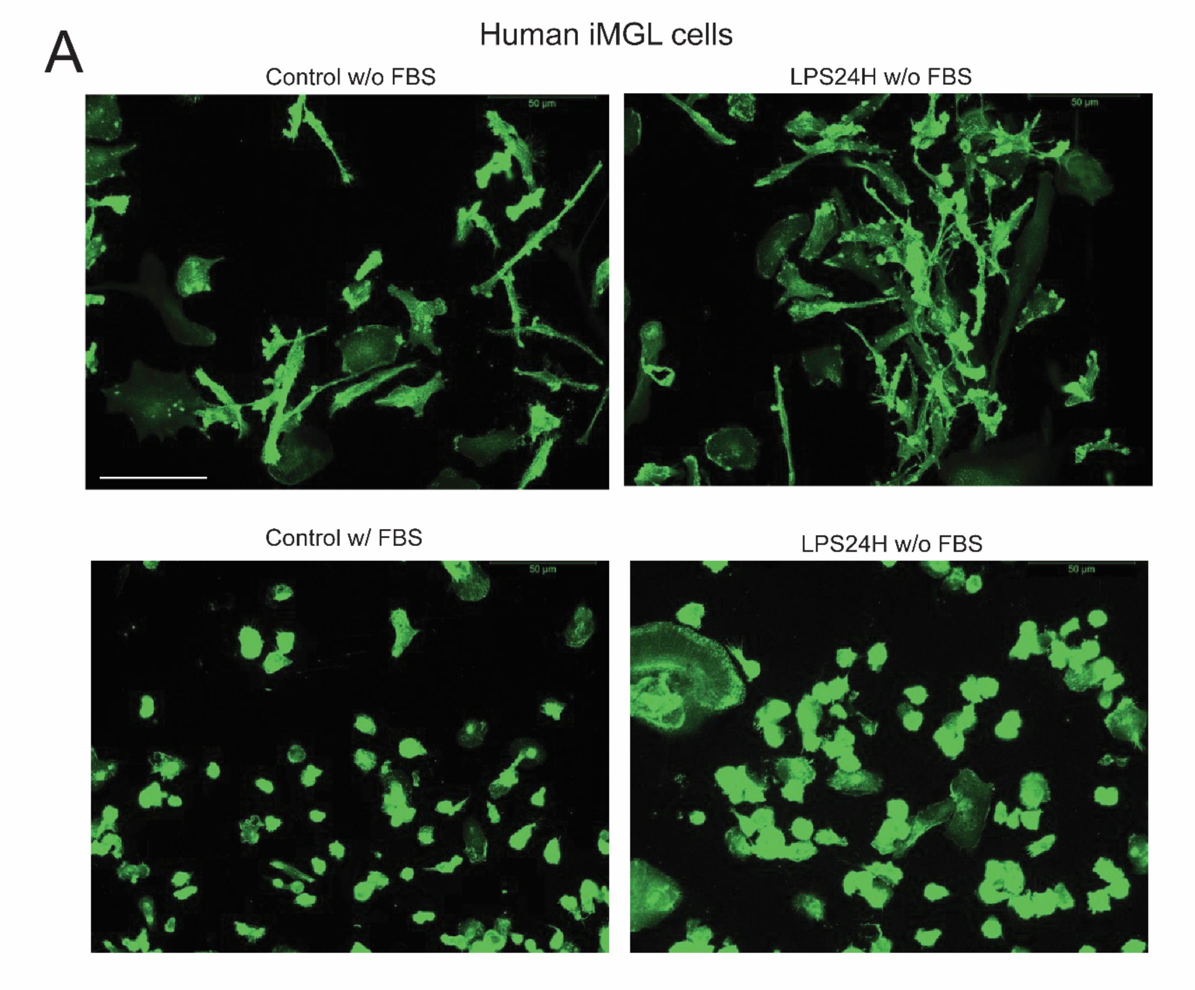

Supplementary Figure 9. Effect of FBS on viability of iMGLs. A. IBA1 immunostaining of untreated and LPS-treated human iMGLs with and without serum. Magnification 20x. Scale bar: 50µm. Representative micrographs of three independent experiments with similar results.

Supplementary Table 1: Key glycolytic enzymes changes upon LPS stimulation in human microglia. Comparison between key glycolytic genes by taking Log2Fold Changes and padjusted values upon LPS stimulation in human microglial models by Alasoo et al., 2015, Hasselman et al., 2019 and the present study.

	Alasoo and colleagues, 2015		Hasselman and colleagues, 2019		This study	
**Gene**	**LPS LOG2FC**	**LPS p adjusted**	**LPS LOG2FC**	**LPS p adjusted**	**LPS LOG2FC**	**LPS p adjusted**
HK1	-0,033945571	0,869757095	0,311869655	0,715240884	-0,449345293	0,000778876
HK2	1,725753088	4,97E-08	0,16974605	0,860490848	0,155577255	0,603020607
HK3	-0,537599882	0,083773302	1,209588858	0,069627517	-0,441352212	0,035084305
PFKL	0,225884443	0,315874198	-0,489771107	0,241800574	0,032290815	0,852953539
PFKM	-0,240184491	0,538907676	0,083985022	0,938397175	-0,64519715	0,065800871
PFKP	0,832655278	0,000560821	0,443239198	0,467758675	0,218589369	0,313727481
PFKFB2	-2,480182269	1,49E-14	-0,172979321	0,805604011	-0,544419049	0,006348326
PFKFB3	3,78475598	2,32E-41	4,205201541	1,89E-10	0,679523678	0,002267515
PFKFB4	0,662009829	0,240592732	1,936316186	0,004624704	-0,908830721	0,038238826

Supplementary Table 2: Key TCA cycle enzymes changes upon LPS stimulation in human microglia. Comparison between key glycolytic genes by taking Log2Fold Changes and padjusted values upon LPS stimulation in human microglial models by Alasoo et al., 2015, Hasselman et al., 2019 and the present study.

	Alasso and colleagues		Hasselman and colleagues		This study	
**Gene**	**LPS LOG2FC**	**LPS p adjusted**	**LPS LOG2FC**	**LPS p adjusted**	**LPS LOG2FC**	**LPS p adjusted**
ACO1	0,421255237	0,025235254	*-0,36264084*	*0,542526946*	*0,697360145*	*0,000652458*
ACO2	-0,197243328	0,42950605	*-0,652235174*	*0,102594347*	*0,083759295*	*0,544608075*
CS	0,015316572	0,929757664	*-0,570141527*	*0,092493501*	*0,291576022*	*0,047077513*
DLST	0,359539115	0,00167459	*0,211144398*	*0,738888198*	*0,133740006*	*0,250510975*
FH	0,008833245	0,971012849	*-0,688039184*	*0,199720543*	*0,422484283*	*0,000382666*
IDH1	-1,266030386	2,29E-05	*0,00116655*	*0,998041115*	*-0,378472565*	*0,000393245*
IDH2	-0,827082955	0,004406717	*-2,069535342*	*3,40E-14*	*-0,34908629*	*0,02583341*
MDH1	-0,376936268	0,205612346	*0,591075912*	*0,560827374*	*0,026806508*	*0,901072063*
MDH2	-0,071247416	0,742852071	*0,154950073*	*0,825393354*	*0,003924168*	*0,983967589*
OGDH	-0,053030142	0,848309813	*-0,891771874*	*0,281191754*	*0,056926869*	*0,768809426*
SUCLG1	-0,370416189	0,063284257	*-0,180815122*	*0,799951978*	*-0,03890903*	*0,871030102*
SUCLA2	-0,193489157	0,159857482	*-0,732743216*	*0,054113095*	*0,038148576*	*0,901142661*

Supplementary Table 3: Primary microglia studies and technical aspects for microglia culture. Columns specify the study, type of culture, if pups or adult mice were used, and whether FBS was used or not.

Study	Microglia culture type	From adult mice or pups?	Was FBS included?
Gosselin et al, 2014	Acutely isolated *ex vivo* , percoll gradient with FACS purification CD11b+ CD45Low	8-9 weeks	Yes
Nike et al., 2012	Acutely isolated ex vivo, percoll gradient	Young (1-2 month old) and Aged (14-16 month old)	Yes
Gosselin et al., 2017	Acutely isolated *ex vivo* , percoll gradient with FACS purification CD11b+ CD45Low	8-9 weeks	Yes
Geric et al., 2019	Brain dissociation and platting with posterior subculturing by mechanical shaking.	Post-natal day 0-1	Yes
Bohlen et al., 2017	Acutely isolated *ex vivo* , myelin depletion and with MACS purification for CD11b+	3-5 weeks	Yes
Chhor et al., 2013	Brain dissociation and platting with posterior subculturing by mechanical shaking.	Post-natal day 0-1	Yes
Dolga et al., 2012	Brain dissociation and platting with posterior subculturing by mechanical shaking.	Post-natal day 1-3	Yes
This study	Brain dissociation and platting with posterior subculturing by mechanical shaking.	Post-natal day 1-3	Yes

Supplementary Table 4: Studies that have stimulated myeloid cells with TLR4 agonism and assessed metabolic outcomes. Columns specify the study, studied species, model, stimuli employed and main metabolic features. (BMDM: Bone marrow-derived macrophages, PBMC: peripheral blood mononuclear cells, BMD: Bone marrow-derived)

Study	Species	Model	Stimuli	Features
Vijayan et al., 2019	Human	hBMDM	LPS	No glycolytic reprogramming<br>No mitochondrial ROS production
Vijayan et al., 2019	Mouse	BMDM	LPS	Mitochondrial dysfunction<br>Glycolytic reprogramming
Malinarich et al., 2015	Human	PBMCs-derived DCs.	LPS	Immature, tolerogenic and LPS-treated tolerogenic DCs were poorly immunogenic and exhibited increased oxidative metabolism.
Verberk et al., 2022	Mouse	BMDM	LPS + IFNγ	Increased glycolysis and decreased oxygen consumption.
Verberk et al., 2022	Human	hMDM	LPS + IFNγ	Increased glycolysis and no effect on oxidative metabolism parameters.
Naler et al., 2022	Mouse	BMD Monocytes	Low dose (100pg/mL)	Enhancers were linked to genes like *Csf1, Irf5, Rab11a, Ccr5, Irf7, Ticam2, Ifit3,* and *Irf1* and with low signal to *Arg1, Hdac5*, and *Treml1*.
Naler et al., 2022	Mouse	BMD Monocytes	High dose (1 µg/mL)	Enhancer-linked genes of autophagy, endocytosis and TLR4 signaling were negatively regulated.
Maoldomhnaigh et al., 2021	Human	hMDM	LPS	Warburg effect occurs rapidly in adult MDM but oxidative metabolism is no longer decreased after 24hr.
Maoldomhnaigh et al., 2021	Human	Umbilical Cord Blood Monocyte Derived Macrophages	LPS	Warburg effect takes longer than in adult MDMs and oxidative metabolism is decreased after 24hr.
