# Supplementary material for: Species-specific metabolic reprogramming in human and mouse microglia during inflammatory pathway induction
Source: Nat Commun. 2023 Oct 13;14:6454. doi: 10.1038/s41467-023-42096-7 (PMC10575978; doi:10.1038/s41467-023-42096-7)
Supplement: Supplementary file 3 — Reporting Summary [file 41467_2023_42096_MOESM3_ESM.pdf]

nature portfolio

Corresponding author(s): NCOMMS-23-04080

Last updated by author(s): September 4th 2023

Reporting Summary

Nature Portfolio wishes to improve the reproducibility of the work that we publish. This form provides structure for consistency and transparency in reporting. For further information on Nature Portfolio policies, see our Editorial Policies and the Editorial Policy Checklist.

Statistics

For all statistical analyses, confirm that the following items are present in the figure legend, table legend, main text, or Methods section.

n/a	Confirmed	
☐	☒	The exact sample size (n) for each experimental group/condition, given as a discrete number and unit of measurement
☐	☒	A statement on whether measurements were taken from distinct samples or whether the same sample was measured repeatedly
☐	☒	The statistical test(s) used AND whether they are one- or two-sided<br>*Only common tests should be described solely by name; describe more complex techniques in the Methods section.*
☐	☒	A description of all covariates tested
☐	☒	A description of any assumptions or corrections, such as tests of normality and adjustment for multiple comparisons
☐	☒	A full description of the statistical parameters including central tendency (e.g. means) or other basic estimates (e.g. regression coefficient) AND variation (e.g. standard deviation) or associated estimates of uncertainty (e.g. confidence intervals)
☐	☒	For null hypothesis testing, the test statistic (e.g. F, t, r) with confidence intervals, effect sizes, degrees of freedom and P value noted<br>*Give P values as exact values whenever suitable.*
☒	☐	For Bayesian analysis, information on the choice of priors and Markov chain Monte Carlo settings
☐	☒	For hierarchical and complex designs, identification of the appropriate level for tests and full reporting of outcomes
☒	☐	Estimates of effect sizes (e.g. Cohen's d, Pearson's r), indicating how they were calculated

Our web collection on statistics for biologists contains articles on many of the points above.

Software and code

Policy information about availability of computer code

Data collection	No custom software was employed for data collection for this study.
Data analysis	Transcriptomic data were analyzed on R, employing the R studio software. Code to perform transcriptomic analyses is saved. Differential expression analysis was performed using the DESEQ2 package.

For manuscripts utilizing custom algorithms or software that are central to the research but not yet described in published literature, software must be made available to editors and reviewers. We strongly encourage code deposition in a community repository (e.g. GitHub). See the Nature Portfolio guidelines for submitting code & software for further information.

Data

Policy information about availability of data

All manuscripts must include a data availability statement. This statement should provide the following information, where applicable:

- Accession codes, unique identifiers, or web links for publicly available datasets
- A description of any restrictions on data availability
- For clinical datasets or third party data, please ensure that the statement adheres to our policy

The datasets generated through (Abud et al., 2017) and re-analyzed for this study are available through GEO Series accession number GSE89189 (https://www.ncbi.nlm.nih.gov/geo/query/acc.cgi?acc=GSE89189).

The datasets generated through (Brownjohn et al., 2018) and re-analyzed for this study are available through GEO Series accession number GSE110952 (https://www.ncbi.nlm.nih.gov/geo/query/acc.cgi?acc=GSE110952).

The datasets generated through (Zhang et al., 2022) and re-analyzed for this study are available through GEO Series accession number GSE175578 (https://www.ncbi.nlm.nih.gov/geo/query/acc.cgi?acc=GSE175578).

We have generated RNAseq data on human and mouse microglia (iPSC-derived microglia and primary microglia) that are available using the accession number GSE221013 (https://www.ncbi.nlm.nih.gov/geo/query/acc.cgi?acc=GSE221013).

The untargeted proteomics data have been deposited to the ProteomeXchange Consortium via the PRIDE partner repository. The dataset identifier is PXD043836 (https://proteomecentral.proteomexchange.org/cgi/GetDataset?ID=PXD043836).

The targeted mass spectrometry proteomics data have been deposited to the ProteomeXchange Consortium via the Peptide Atlas. The dataset identifier PASS05835 (https://db.systemsbiology.net/sbeams/cgi/PeptideAtlas/PASS_View?identifier=PASS05835).

Source data are provided with this paper.

Research involving human participants, their data, or biological material

Policy information about studies with human participants or human data. See also policy information about sex, gender (identity/presentation), and sexual orientation and race, ethnicity and racism.

Reporting on sex and gender	NA
Reporting on race, ethnicity, or other socially relevant groupings	NA
Population characteristics	NA
Recruitment	NA
Ethics oversight	NA

Note that full information on the approval of the study protocol must also be provided in the manuscript.

Field-specific reporting

Please select the one below that is the best fit for your research. If you are not sure, read the appropriate sections before making your selection.

- [x] Life sciences
- [ ] Behavioural & social sciences
- [ ] Ecological, evolutionary & environmental sciences

For a reference copy of the document with all sections, see nature.com/documents/nr-reporting-summary-flat.pdf

Life sciences study design

All studies must disclose on these points even when the disclosure is negative.

Sample size	For cell-based assays, no sample-size calculation were performed. For transcriptomic and proteomic analysis, no sample-size calculation were performed and this number of replicates was selected because at least three biologically independent replicates have typically been shown to be sufficient for robust detection of differentially expressed genes in related studies.
Data exclusions	Initially, a condition of LPS+DCA was included in the RNA sequencing run. After inspecting the transcriptome of these samples, we decided to not include them in the manuscript due to that they were virtually identical to the LPS condition. We believe the DCA concentration was suoboptimal.
Replication	Cell-based experiments were replicated in separate set ups with different animals, or differentiations.
Randomization	Cell-based experiments were performed as batches, where all conditions were tested per replication. iPSC-derived microglial differentiation exhibited a batch effect observable by principal component analysis. Given this, batch number was included as a covariate in the design formula of the DESEQ2 pipeline which was used for modeling.
Blinding	Blinding was not relevant for our study.

Reporting for specific materials, systems and methods

We require information from authors about some types of materials, experimental systems and methods used in many studies. Here, indicate whether each material, system or method listed is relevant to your study. If you are not sure if a list item applies to your research, read the appropriate section before selecting a response.

Materials & experimental systems

n/a	Involved in the study
☐	☒ Antibodies
☐	☒ Eukaryotic cell lines
☒	☐ Palaeontology and archaeology
☐	☒ Animals and other organisms
☒	☐ Clinical data
☒	☐ Dual use research of concern
☐	☐ Plants

Methods

n/a	Involved in the study
☒	☐ ChIP-seq
☒	☐ Flow cytometry
☒	☐ MRI-based neuroimaging

Antibodies

Antibodies used	IBA1 Rabbit (Wako 019-19741) Lot number: PTR2404<br>Alexa Fluor 488 (Invitrogen A11034) Lot number: 2256732
Validation	For the primary antibodies we have relied on validation by supplier.

Eukaryotic cell lines

Policy information about cell lines and Sex and Gender in Research

Cell line source(s)	The induced pluripotent stem cell (iPSC) cell line Ctr8.2 (UEF-2B) was obtained from Virtanen Institute for Molecular Sciences - University of Eastern Finland (Holmqvist et al., 2016). Another iPSC line purchased from Gibco was also used for the functional assays (Seahorse measurements).
Authentication	Cells were authenticated prior to acquisition.
Mycoplasma contamination	Cells were negative for mycoplasma contamination.
Commonly misidentified lines (See ICLAC register)	None.

Animals and other research organisms

Policy information about studies involving animals; ARRIVE guidelines recommended for reporting animal research, and Sex and Gender in Research

Laboratory animals	Mus musculus (C57BL/6J Mice) of postnatal days 0-3 were employed. Pregnant females (3 to 6 months) were maintained on a 12:12h dark:light cycle and received food and water ad libitum.
Wild animals	The study did not involve wild animals.
Reporting on sex	Sex-based reporting was not performed in our study. Sex of mice employed for primary microglial cultures was not reported.
Field-collected samples	Our study did not involve collection of samples from the field.
Ethics oversight	C57BL/6 J mice from the central animal laboratory at University of Groningen were housed and handled in accordance to Dutch standards and guidelines (Protocol 171224-01-003). All experiments were approved by the University of Groningen Committee for Animal Experimentatio

Note that full information on the approval of the study protocol must also be provided in the manuscript.
