# Supplementary material for: Species-specific metabolic reprogramming in human and mouse microglia during inflammatory pathway induction
Source: Nat Commun. 2023 Oct 13;14:6454. doi: 10.1038/s41467-023-42096-7 (PMC10575978; doi:10.1038/s41467-023-42096-7)
Supplement: Supplementary file 4 — Description of Additional Supplementary Files [file 41467_2023_42096_MOESM4_ESM.pdf]

Description of Additional Supplementary Files

Supplementary Dataset 1: Differential gene expression comparison between LPS4 hours versus control in Murine microglia. Columns depict output of DESEQ2 results function: Gene name, mean expression of the control condition, Log2FoldChange of the difference between conditions, lfcSE (Standard Error of the Log2FoldChange), stat value (Wald statistic), pvalue and adjusted pvalue (multitesting correction).

Supplementary Dataset 2 Differential gene expression comparison between LPS24 hours versus control in Murine microglia. Columns depict output of DESEQ2 results function: Gene name, mean expression of the control condition, Log2FoldChange of the difference between conditions, lfcSE (Standard Error of the Log2FoldChange), stat value (Wald statistic), pvalue and adjusted pvalue (multitesting correction).

Supplementary Dataset 3 Differentially expressed genes in LPS-treated murine microglia. Left table depicts filtering of genes with padjusted values less than 0,05 in the comparisons of 4 and 24 hours LPS versus control. Right table depicts filtering of genes with padjusted values less than 0,05 and absolute Log2Fold Changes of at least 1,5 in the comparisons of 4 and 24 hours LPS versus control.

Supplementary Dataset 4 Differential gene expression comparison between LPS24 hours versus LPS4 hours in Murine microglia. Columns depict output of DESEQ2 results function: Gene name, mean expression of the control condition, Log2FoldChange of the difference between conditions, lfcSE (Standard Error of the Log2FoldChange), stat value (Wald statistic), pvalue and adjusted pvalue (multitesting correction).

Supplementary Dataset 5. Differential gene expression comparison between LPS4 hours versus Control in Human microglia. Columns depict output of DESEQ2 results function: Gene name, mean expression of the control condition, Log2FoldChange of the difference between conditions, lfcSE (Standard Error of the Log2FoldChange), stat value (Wald statistic), pvalue and adjusted pvalue (multitesting correction).

Supplementary Dataset 6. Differential gene expression comparison between LPS24 hours versus Control in Human microglia. Columns depict output of DESEQ2 results function: Gene name, mean expression of the control condition, Log2FoldChange of the difference between conditions, lfcSE (Standard Error of the Log2FoldChange), stat value (Wald statistic), pvalue and adjusted pvalue (multitesting correction).

Supplementary Dataset 7. Differential gene expression comparison between LPS24 hours versus LPS4 hours in Human microglia. Columns depict output of DESEQ2 results function: Gene name, mean expression of the control condition, Log2FoldChange of the difference between conditions, lfcSE (Standard Error of the Log2FoldChange), stat value (Wald statistic), pvalue and adjusted pvalue (multitesting correction).
